# Observations on the Surgery of the Ancients, Vindicating Their Claims to Many of the Reputed Discoveries and Improvements of Modern Times

**Published:** 1814-10

**Authors:** David Hosack

**Affiliations:** Professor of the Theory and Practice of Physic and Clinical Medicine in the University of the State of New-York, Member of the American Philosophical Society, of the New-York Historical Society, &c.


### *For the Medical and Physical Journal.*

*Observations on the Surgery of the Ancients, vindicating their Claims to many of the reputed Discoveries and Improvements of modern Times.* By DAVID HOSACK, M.D. Professor of the Theory and Practice of Physic and Clinical Medicine in the University of the State of New-York, Member of the American Philosophical Society, of the New-York Historical Society, &c.

(*Concluded from p.* 119.)

THE practice of Celsus is, at present, pursued, under similar circumstances, by that celebrated surgeon, Mr. Abernethy, of St. Bartholomew's hospital; who has, in numerous cases, dispensed with the trephine, having relieved his patient by the application of other remedies. But, if the symptoms became more formidable, and the patient experienced no benefit from the first operation, Celsus then directed such portions of the bone to be removed, as the extent of the injury appeared to require. In those cases where the fracture or depression was small, he employed the instrument called the *modiolus;* which, in its construction, is similar to the trephine. But when the fracture was extensive, or of an irregular shape, he made use of a perforator, so as to surround the part affected with holes; and afterwards employed the chisel and mallet to cut out the portion of bone inclosed within them. In this operation, large portions were removed, which the modiolus could not cover, unless frequently applied. Mr. Hey, of Leeds, has lately introduced a substitute for the trephine; by which the same advantages are obtained as by the perforator and chisel of Celsus.

As anatomy in those days was very imperfectly understood, we cannot be surprised that Celsus was less successful in some of the more complicated operations of surgery. Accordingly, in reducing strangulated hernia, he experienced great embarrassment: but it is to be recollected, that this operation requires an accurate knowledge of the structure of the parts concerned, to guide the knife of the operator. This knowledge has only been acquired within a few years. In the hands of John and Charles Bell, Mr. Astley Cooper, of London, and Mr. Hey, of Leeds, this operation has received its best and most important improvements. But in the history given by Celsus, of the symptoms and causes of hernia, and of the nature of its contents, he is no less perspicuous than upon most other subjects that came under his notice. He describes, with great accuracy, most of the species of hernia now found in books of surgery, except such as occur under

Poupart's ligament, and that which takes place between the abdominal muscles. The umbilical hernia he distinguishes into the omental and intestinal, according as the omentum or intestine is contained in the hernial sac. In like manner he describes the scrotal hernia, as containing omentum, intestine, or both combined, with the symptoms which characterize each species.

He also distinguishes scrotal rupture, as it occurs in the young child and in the adult. In the former, he directs a compress to be applied to the part, and retained by means of a roller; by which application, he observes, the intestine is oftentimes forced in, and the coats agglutinated: but in the adult, when the intestine is protruded in a large quantity, and attended with symptoms of danger, it appears that he was unacquainted with the mode of replacing it within the abdomen. He observes, it can only be reduced as far as the groin; thereby producing a change, but not a termination of the disease: and in those cases where he considered an operation necessary, he did not restore the displaced intestine, but by means of a ligature or the knife he diminished the quantity of loose skin, and by the inflammation and thickening of the parts induced, formed a cicatrix, which was calculated to prevent an increase of the prolapsus. Upon the same principle, in umbilical hernia, when the parts protruded were restored to their natural situation, he excited inflammation by ligature and caustics; which so changed the structure of the parts, as to prevent a return of the rupture. Thus far his operation was successful; but in some instances, from want of anatomical knowledge, he considered it necessary to remove parts which have no connection with the disease. In hernia of the intestine, he sometimes removed not only a portion of the hernial sac, but also extirpated the testicle itself.

In hernia of the omentum, if small, he replaced it by the hand alone; and to prevent it from being again protruded, he applied a compress to the part, and secured it by a bandage passed round the loins; but if large, he did not attempt to reduce it. He then, to effect a separation of the mortified parts, made use of escharotics or ligatures, preferring them to the scissors or knife, on account of the hemorrhage which attends the use of cutting instruments. The same observation is made, and a similar practice adopted by Mr. Hey, of Leeds.

Celsus also performed many other operations in diseases of the abdomen and pelvis, including the organs of generation. In wounds of the intestines, he performed the operation of gastroraphy; and his description of the mode of performing forming it, corresponds with the directions given by the best writers of the present time. In the treatment of dropsy, he performed the operation of tapping, not only on the side of the belly, but, in some instances, he perforated the navel; which is considered, at this day, as the latest improvement in the manner of performing paracentesis. The hydrocele, circocele, and the inflammation of the testicle, are also well characterised by this accurate observer. He cured hydrocele by incision. The varix of the spermatic cord, as well as the varices of the veins of the legs, he cured by laying bare the vein thus enlarged, and by applying the actual cautery; or removed it by excision, having previously separated the vein, and inclosed it with ligatures, above and below the part to be removed. He divided the prepuce in phymosis; he introduced the catheter in retention of urine, both in males and females. Celsus also performed the important operation of lithotomy; but considered it as attended with great danger. He very judiciously prepared his patient for this, as for every other capital operation, by abstinence from solid food and stimulating drinks. His mode of operating was, in some respects, peculiar to himself, and was hence denominated *lithotomia Celsiana;* but, in most circumstances, it agreed with that afterwards adopted by Paulus Ægineta, and others, by the apparatus minor, or cutting on the gripe. When the operation was finished, he bled his patient, enjoined abstinence, made use of a warm bath, and oily fomentations, to diminish inflammation: but he advised the operation to be performed only in the spring of the year, and confined it to patients between nine and fourteen years of age.

These facts teach us, that Celsus was not sufficiently acquainted with anatomy to perform this operation, without great hazard to the patient. At this we cannot be surprised, when we recollect that it is only within a few years that it has been performed with general success. Our only surprise should be, that the art of surgery was so well understood as it appears to have been in his day. Among other operations, he extracted the dead fœtus from the womb, by means of the crotchet; and, when difficulty presented, he divided the child, and removed it piece-meal. To restrain hemorrhage, or to remove any inflammation induced by the operation, he applied soft cloths, wet with an infusion of vinegar and roses. Condylomata (or tubercles of the anus) and hemorrhoidal tumours, he removed with the knife and ligatures. The same treatment you will find adopted and recommended by Mr. Ware, in his valuable essay on that subject; but which contains no reference to the practice of Celsus. Fistula in ano he also cured by ligature, making making use of a linen thread instead of the leaden wire employed by the moderns. This mode of treating fistula was revived by Foubert, and has been frequently practised in France since that time. Professor Camper has also made use of ligatures, in those whom he found fearful of the knife, or when the patient could not submit to the necessary confinement which the incision requires. But the great inconvenience which arises from the long-continued irritation of the ligature, and the prolapsus ani which it sometimes produces, have occasioned it to be laid aside.

Celsus was no less successful in the treatment of fractures and dislocations. His directions for reducing the fracture of the clavicle, and his treatment of fracture of the ribs, correspond with those contained in the present systems of surgery; and in fractures of the extremities, although he did not employ the many-tailed bandage, or place the broken limb in a flexed position, but confined it to the fracture-box, he remarks, that the smaller bones were generally united between fourteen and twenty-one days; those of the leg and fore-arm, between twenty and thirty; and those of the arm and thigh-bone, between twenty-seven and forty days. Few surgeons of the present time, I believe, can boast of greater success.

An eminent modern physician emphatically exhorts every person in the study of medicine to keep Celsus in his hands by night and by day. I trust the outlines I have exhibited of his practice of surgery, and the evidences I have adduced of his skill in that art, will also induce the pupil in *surgery* to give his writings an attentive perusal.

Galen, the physician of the Emperor Marcus Aurelius, also holds a distinguished place in the history of surgery. He was born at Pergamus, a city of Asia, about 130 years after Christ, and during the reign of the Emperor Adrian. He was celebrated not only as a practitioner in physic and surgery, but as the most accurate anatomist that had then appeared. His knowledge of anatomy, at the same time that it furnished him with more correct views of the functions of the human body, and of the general principles of surgery, also enabled him to perform some operations unknown to his predecessors. By some who profess to give an account of his works, he is considered as the mere commentator on the writings of Hippocrates, especially upon those subjects which relate to surgery. But although Galen selected every thing he considered valuable in the works of those who had gone before him, he has left the evidence of great original genius, not only as a physician, but as a practitioner of surgery. I may remark, that his writings, like like those of Celsus, have been plundered by the moderns, without the least acknowledgment of the source whence they derived the materials of what they afterwards denominated discoveries and improvements.

Without going into a detail of the practice of Galen, I shall confine my remarks to a few of those subjects in which he appears to have added to the stock of knowledge he had received from his predecessors.

In his chapter on ulcers, he distinguishes their several species with so much correctness, that it has evidently been the basis of Mr. Benjamin Bell's treatise upon this subject. Not only the names by which they were designated by Galen are retained, but even his definition of an ulcer has been adopted by our late systematic. "A solution of continuity, in any of the softer parts of the body," is the definition of an ulcer given by Mr. Bell. "Unitatis solutio est in carnosa parte," is the language of Galen. I may also add, that, in the treatment of ulcers, there is scarcely what is called an improvement in the practice of the moderns that was not known to Galen. During the inflammatory stage of phlegmon, he carefully enjoined his patient to abstain from wine and stimulants generally. He also made use of the lancet, purgatives, and warm bathing, to diminish the inflammation; but, when the ulcer was formed, and a free discharge of matter obtained, he directs his remedies according to the character of the wound. In the simple purulent ulcer, his practice was to bring the edges as nearly as possible into contact. In the sordid ulcer, he made use of honey, verdigris, and terebinthinate applications, to change its condition, and to promote the growth of healthy parts. Oily dressings, and relaxing cataplasms, he very properly proscribed, as injurious in those cases. With the same view of restoring a healthy action in the part, he directs it to be washed with wine; and, as an evidence that he had in view its stimulant qualities, he observes, that the Falernian, which was a sweet wine, was useless in this respect. "Nam quecunque dulcia pariter et fulva sunt, ut Falernum, ad id inutilia existunt."

But one of the most approved modes of treating ulcers, in the present day, is by bandage. This also was the practice of Galen; and in his direction upon the application of the roller, he observes, it should not be so loose as to give no support to the limb, nor bound so tight as to excite pain by its pressure. "Circumductio etiam ipsa non ita laxa sit ut nihil efficiat nec ita vehemens ut dolorem premendo excitet." In punctures of the nerves, the practice of Galen was also original. If the wound be large, he advises it to be kept open; but, if small, he directs it to be dilated, and terebinthinate and other stimulating dressings to be applied, for the purpose of obtaining a free discharge from the surface of the wound. "Ubi aliquis nervus est punctus, cutem ipsam servari oportet, aut quod tutius est latius incidere." In warm climates, the same practice is successfully pursued under similar circumstances, in the present day.

For the purpose of suppressing hemorrhage, when the external application of cold water and astringents failed, such as unripe galls, balaustines, and the stronger austere wines, it was the practice of Galen to raise the bleeding vessel, by means of a hook, and to secure it by a vinculum or ligature. Ambrose Paré, in the sixteenth century, therefore unjustly claimed the use of the ligature as his discovery.

The aneurismal tumour is also well characterized in the writings of this celebrated surgeon. From some expressions contained in his work, I am also induced to believe, that in the treatment of fractures he placed the broken limb in a flexed position. It was certainly his practice, when the bones were secured by bandage, to place the limb in that situation which occasioned the least pain to his patient; and in the application of the bandage, he was aware of the inconvenience and injury arising from too severe pressure upon the part affected. To aid the generation of bony matter, in forming a callus, he also, as in wounds of the soft parts, made use of wines and other stimulating applications.

Two other writers of respectability among the ancients merit our attention, Ætius and Paulus Ægineta. I pass over the works of Oribasius, as they contain nothing important, but what is to be found in the writings of Galen.

Ætius flourished at the end of the fifth and beginning of the sixth century. He was born at Amida, in Mesopotamia, and was educated at the celebrated medical school of Alexandria. Although his works are not so well digested or arranged as those of Paulus, his chirurgical writings contain many valuable observations, not to be found in Celsus or Galen, and were even omitted by his successor Paulus. His account of the diseases of the eyes, is much more minute and complete than the chapter of Celsus upon the same subject. In the treatment of anasarca, he prescribes scarification of the extremities; not merely for the purpose of a temporary evacuation of the water contained in the cellular membrane, as had been recommended by Hippocrates, but made his incisions so bold and extensive, that he thereby, according to his own observation, not only relieved his patient of anasarca, but frequently cured him of ascites. Sylvius de la Boe afterwards wards proposed to perform this species of tapping by means of a needle, and claimed the discovery as his own.

I have already remarked, that Hippocrates and Celsus made frequent use of caustics, especially in the treatment of dropsy, epilepsy, sciatica, and phthisis. Ætius also employed them, not only in the same complaints, but in many other diseases. In asthma, palsy, empyema, and affections of the head; in obstinate head-aches, or in injuries of the brain, he applied them to the nape of the neck, and to different parts of the head. He directed them to be long-continued, to be applied in great numbers, and made of a circular shape, that they might be slow to heal, and thereby afford a large discharge. In the bite of a mad dog, he directed them to be kept open forty or sixty days; and if, in that time, they should, by accident, be closed, he renewed their application. In this free use of caustics, he was followed by Paulus. The issues, as employed at this day, are found useful in the same diseases, for which the caustics were so successfully applied by the ancients. It is said by some, that the use of the *seton* was known to Ætius: this does not appear. Roland, of the tenth century, was the first who described this species of issue. It was afterwards spoken of by Rhazes and Albucasis, as much in use in their day. Dr. Freind remarks, that whoever reads the chapter of Rhazes on this subject, will find that the ancients understood the value of this remedy as well as the moderns.

Ætius also treated of the diseases of women. He notices the causes of difficult labour, and directs the mode of delivery, which should accordingly be pursued. In one respect, his work is imperfect; as he takes no notice of an important branch of surgery, fractures and dislocations.

Paulus Ægineta, so called from his birth-place, the island Ægina, flourished about the middle of the seventh century. He also was a pupil of the Alexandrian school. After finishing his education, he travelled into different countries, and thereby had more extensive opportunities of becoming acquainted with diseases than most of those who had preceded him. His works, accordingly, contain much original matter. His chirurgical writings, in which he devoted a book exclusively to the operations of surgery, have been universally considered as more complete than any that appeared before the revival of learning in the fifteenth century. It is sufficient evidence of the value of the works of Paulus, that his writings, with those of Celsus, became the text books of Fabricius, a celebrated surgeon of the sixteenth century.

In his treatise on ruptures, the different species of hernia are more minutely detailed, and the operation more circumstantially stantially described than by Celsus. That species of aneurism, arising from a wound or rupture of an artery, which was known to Galen, he describes with great accuracy, as well as the circumstances by which it is distinguished from other tumours. This is not all. In the treatment of the disease, he performed the operation for its removal, in the same manner as is done at this day, by securing the vessel above and below the part affected, and dividing it between the ligatures. He also describes the fracture of the patella, which was not noticed before his time. In chronic affections of the head, and in diseases of the eyes, he opened the jugular veins; and, in some instances, the arteries behind the ears. To render the operation of cupping more effectual, he improved the scarificator in such manner as to make several incisions at the same time. In cases of quinsy, threatening suffocation, he performed the operation of bronchotomy. He directs it to be performed about the third or fourth ring of the trachea, being a part which he observes is less covered with flesh, and where there is the least danger of dividing many vessels. He is also careful not to make the aperture larger than is sufficient for the purpose of respiration. In this operation, Albucasis afterwards copied Paulus, without an acknowledgment.

Paulus also improved much upon his predecessors, in the manner of performing some of the more usual operations of surgery. In extracting the stone from the bladder, he did not, like Celsus, confine the operation to childhood, but performed it at any period of life. He also directs the incision to be made, not in the middle of the perineum, as was done by Celsus, but upon the left side of it, nearly where the incision for the lateral operation is at this day made. Upon this subject he also gives another important direction; to make the external incision free and large, by which both the sufferings of the patient and the danger of the operation are diminished. Paulus also treats of the diseases of pregnancy, and was skilled in the practice of midwifery.

Such was the state of surgery among the ancients; and from the progress they had made, much also was to be expected from the labours of their successors: but a long interval of ignorance and darkness now ensues.

The civilized parts of Asia and Africa were overrun by the Saracens, under Mahomet and his successors. Being, from their religious tenets, the professed enemies to all knowledge not contained in the Koran, they ordered the celebrated library of Alexandria to be destroyed; and with it all the liberal arts had nearly perished.

About the same period, the Goths and Vandals overran the the Roman empire. They also were the enemies of science, because they were strangers to it. But after the fury of the Saracens had somewhat subsided, the love of health and life, which is natural to man, induced them to revive the healing art, which had in a great degree shared the general fate of learning. The works of Hippocrates and Galen were sought for, and translated first into the Syrian, and thence into the Arabian language: in a few centuries, the Mahomedan governments abounded in schools of physic. Although the works of the most celebrated Greek and Roman writers were transcribed by the Arabian physicians Rhazes and Avicenna, the science of medicine received little improvement from their hands. In like manner, the knowledge of surgery, among the Arabians, was preserved, but not improved. The precept of Mahomet, which forbad the opening of dead bodies, must necessarily have prevented improvements in anatomy, and consequently retarded their progress in the practice of surgery. Notwithstanding the labours of Rhazes, Avicenna, Albucasis, and other eminent practitioners and teachers of medicine among the eastern nations, surgery received few or no additions, from the time of Paulus until the sixteenth century; when Fabricius, of Aquapendens, published his celebrated system, containing, not only the surgery of the ancients, but many original observations, which may be perused with much interest, even at this day. Of Fabricius, Boerhaave observes, "ille superavit omnes;" "omnibus potius quam hocce carere possumus."

Upon some future occasion, I propose to take a view of the progress of this art, from the revival of learning to the present period; in which I shall enumerate the advantages which the practice of surgery has derived from the discovery of the circulation of the blood, and the subsequent improvements in anatomy and the other branches of the healing art. D. HOSACK.

